# Guardians of the Gut – Murine Intestinal Macrophages and Dendritic Cells

**DOI:** 10.3389/fimmu.2015.00254

**Published:** 2015-06-02

**Authors:** Mor Gross, Tomer-Meir Salame, Steffen Jung

**Affiliations:** ^1^Department of Immunology, Weizmann Institute of Science, Rehovot, Israel; ^2^Biological Services, Weizmann Institute of Science, Rehovot, Israel

**Keywords:** gut, dendritic cells, macrophages, homeostasis, inflammation, IBD

## Abstract

Intestinal mononuclear phagocytes find themselves in a unique environment, most prominently characterized by its constant exposure to commensal microbiota and food antigens. This anatomic setting has resulted in a number of specializations of the intestinal mononuclear phagocyte compartment that collectively contribute the unique steady state immune landscape of the healthy gut, including homeostatic innate lymphoid cells, B, and T cell compartments. As in other organs, macrophages and dendritic cells (DCs) orchestrate in addition the immune defense against pathogens, both in lymph nodes and mucosa-associated lymphoid tissue. Here, we will discuss origins and functions of intestinal DCs and macrophages and their respective subsets, focusing largely on the mouse and cells residing in the lamina propria.

## The Unique Characteristics of the Gut Landscape

Intestinal mononuclear phagocytes are located in a unique anatomic environment that necessitated the evolution of special functional adaptations of these cells. Exposure to commensal bacteria and harmful pathogens, as well as nutrients and food antigens, in the intestinal lumen force the immune system to continuously weigh tolerogenic and protective immune response. Disruption of this critical and delicate balance can result in devastating inflammatory reactions, e.g., hyper-reactivity to food components (1) or inflammatory bowel diseases (IBD), such as Crohn’s disease or ulcerative colitis (2).

Both dendritic cells (DC) and macrophages are found spread throughout the connective tissue that underlies the epithelial layer of the gut, the lamina propria. Moreover, representatives of the two main mononuclear phagocyte families are also located in mucosa-associated lymphoid tissue (MALT), including Peyers’ Patches and isolated lymphoid follicles (ILFs) (3). DC and macrophages have distinct, yet complementary roles in maintaining gut homeostasis and immune defense. In keeping with their migratory capacity, DC translocate from the lamina propria via the lymphatics to the gut-draining mesenteric lymph nodes (MsnLNs), where they present antigens to naïve T cells, polarize them toward effector fates, and thus establish the adaptive branch of the immune system (4).

Macrophages, on the other hand, are believed to contribute to the local clearance of bacteria from the tissue, translate alert signals to other immune cells, secrete cytokines to establish the local homeostatic immune cell network, and participate in T cell re-stimulation and maintenance within the lamina propria (5).

DC and macrophages can, as discussed in detail below, be divided into several subpopulations with defined origins, overlapping and distinct surface marker profiles, functions and locations. The best characterized DC and macrophage subsets and their key features are summarized in Table 1.

**Table 1:** **Mononuclear phagocytes and their respective subsets in the lamina propria of the mouse intestine**.

Intestinal mononuclear phagocyte	Main markers (additional markers)	Location	Precursor	Growth/transcription/environmental factor dependence	Functional specialization	Additional comments	Selected references SI, LI indicate organ of study: small or large intestine
DC	CD103+ CD11b− (CD24+, XCR1+)	Lamina propria, MALT	preDC	Flt-3L Irf8, Id2, Batf-3	Cross-presentation	Equivalent of splenic XCR1+ CD8a+ DC	Edelson et al. (6) SI
							Ginhoux et al. (7) SI
							Becker et al. (8) SI
							Crozat et al. (9) SI
							Schlitzer et al. (10) SI
	CD103+ CD11b+ (CD24+, Sirpα+)	Lamina propria, MALT	preDC	Flt-3L (partially) Csf-2 (GM-CSF), Irf-4, Notch2, Retinoic acid (ileum)	Required for generation and priming of TH17 cells	More prevalent in ileum	Bogunovic et al. (11) SI, LI
							Lewis et al. (12) SI, LI
							Welty et al. (13) SI, LI
							Schlitzer et al. (10) SI
							Persson et al. (14) SI, LI
							Klebanoff et al. (15) SI
	CD103− CD11b+		preDC	Flt-3L, Csf-1 (M-CSF)	Priming of IL-17 and INFγ-producing T cells		Bogunovic et al. (11) SI, LI Cerovic et al. (16) SI Scott et al. (17) SI, LI
	CD103− CD11b-		preDC	Ftl3L	Priming of TH17 *(in vitro)*		Cerovic et al. (16)
Macrophages	CD64+ CX3CR1+ CD11c+ (F4/80+ CD11b+)	Lamina propria	Ly6C+ monocytes	Csf-1 (M-CSF) Csf-2 (GM-CSF) (in colon)			Niess et al. (18) SI
							Varol et al. (19) SI
							Bogunovic et al. (11) SI
							Mortha et al. (20)
							Cecchini et al. (38)
	CD64+ CX3CR1+ CD11c− (F4/80+ CD11b+)	Lamina propria	Ly6C+ monocytes	Csf-1 (M-CSF) Notch 1/2			Ishifune et al. (21) SI Cecchini et al. (38), SI LI
	CD64+ CX3CR1+ CD169+ (F4/80+ CD11b+)	Crypt proximity	Ly6C+ monocytes	Csf-1 (M-CSF)			Hiemstra et al. (22) LI Cecchini et al. (38), SI LI
	CD64+ CX3CR1+ (F4/80+ CD11b+)	Muscularis layer	Ly6C+ monocytes	Csf-1 (M-CSF)	Communication with neurons		Muller et al. (23) SI, LI Cecchini et al. (38), SI LI

With this review, we provide an overview on the characteristics and function of intestinal macrophages and DC in the mouse, including specific roles of their subpopulations. We will discuss distinct origins, roles in maintaining gut homeostasis, and the interactions between these cells and other immune cells. Finally, we will review their communication with their non-immune microenvironment and elaborate on emerging roles of macrophages and DC in inflammation.

## Intestinal Macrophages

Macrophages are the most abundant mononuclear phagocytes in the steady-state gut lamina propria (3, 24). Intestinal macrophages are currently best characterized by their expression of CD64, the Fcγ receptor 1 (FcγRI) (25), and the chemokine receptor CX_3_CR1 (18), as well as the F4/80 antigen (EGF-like module containing mucin-like hormone receptor-like 1-EMR1) and the integrins CD11b and CD11c (26). Due to the high surface expression levels of the chemokine receptor CX_3_CR1 by gut macrophages, these cells can also be readily detected, isolated, and studied *in situ* using intra-vital microscopy on mice harboring a GFP reporter gene inserted into the CX_3_CR1 locus (27).

## Ontogeny

Like other tissue macrophages (28), also intestinal macrophages are first established before birth from precursors originating in the yolk sac or fetal liver (29). However, unlike macrophages in most other tissues, these embryo-derived cells are replaced in the gut shortly after birth by cells that derive from Ly6C^+^ blood monocytes (29). The adult monocyte-derived cells display a uniquely short half-life for macrophages (30) indicating their continuous renewal. The monocytic origin of intestinal macrophages was first established in adoptive transfer experiments, involving the transfer of CX3CR1^gfp^ monocyte-precursors and monocytes into CD11c-DTR transgenic mice, whose CD11c-expressing cells, including intestinal macrophages, were depleted by a diphtheria toxin challenge (11, 19, 31). During their differentiation into gut macrophages, monocytes lose Ly6C expression, while other surface markers, such as MHCII, F4/80, CD64, CD11c, and CX3CR1 are up-regulated (25, 32, 33). Moreover, the cells acquire a characteristic anti-inflammatory gene expression profile (32, 34), whose timely establishment and maintenance are critical for gut homeostasis (35). This includes the expression of IL-10, TREM-2, IRAK-M, and tumor necrosis factor (TNF)AIP3 genes, but also of TNFα, which has both pro- and -anti-inflammatory activity (32). Of note, this expression profile is robust, as it seems to withstand acute challenges, such as the ones associated with oral dextran sulfate sodium (DSS) exposure (32). The molecular cues that drive the “education” of the macrophages in various regions of the gut remain to be defined, but the epithelium is likely to play a role in this process. Epithelial cells could control macrophage differentiation by secretion of immune-regulatory factors, such as thymic stromal lymphopoietin (TSLP), transforming growth factor-β (TGF-β), and prostaglandin E-2 (PGE-2) (36). In addition, recent findings suggested that semaphorin 7A, which is secreted by epithelial cells, contributes to the induction of IL-10 expression by CX_3_CR1^+^ intestinal macrophages (37). Also, colony-stimulating factor 2 (Csf-1; previously named macrophage colony-stimulating factor, M-CSF) and colony-stimulating factor 2 (Csf-2; previously named granulocyte-macrophage colony-stimulating factor, GM-CSF) play a role in the development of macrophages. Csf-1 is a crucial factor for monocyte development, as Csf-1-deficient osteopetrotic (op/op) mice display reduced levels of F4/80^+^ cells in the small and large intestine after the first few days of life (28, 38, 39). Csf-2-depleted mice were shown have reduced numbers of CD11c^+^ colonic macrophages (20).

Of note, Ly6C^+^ monocytes fail to acquire the characteristic macrophage quiescence during intestinal inflammation, but under this condition respond to local factors that trigger pattern recognition receptors, such as TLRs and NLRs, giving rise to pro-inflammatory macrophages (32). These pro-inflammatory cells, which in acute inflammation outnumber the resident macrophage population, secrete IL-12, IL-23, TNF-α, and inducible nitric oxide synthase (iNOS) (32).

A key suppressor of macrophage-associated inflammation is the IL-10/IL-10 receptor (IL-10R) axis, as mice bearing mutations in IL10-Ra in intestinal CX3CR1^+^ macrophages developed severe colitis (35) comparable to the pathology reported for IL-10-deficient animals (40). This central critical role of IL-10 in maintaining the non-inflammatory state of macrophages, and thereby, gut homeostasis is also supported by research conducted on samples from humans with loss of function mutations in IL-10R (41). The latter provides an explanation for the severe early onset of colitis observed in pediatric patients harboring nonsense and missense mutations in IL-10R, which reduce IL-10R expression and hamper its signaling cascades (42). Interestingly though, IL-10 production by intestinal macrophages, although also prominent, seems to be redundant for the maintenance of gut homeostasis (35); rather the system seems to rely on alternative IL-10 sources, such as Treg cells (43).

Homeostatic monocyte recruitment to the gut is thought to depend on the chemokine receptor CCR2, as CCR2-deficient mice display less intestinal macrophages and CCR2-deficient intestinal macrophages are underrepresented in mixed bone marrow chimeras (24, 25). The exact factors and mechanisms that ensure homeostatic Ly6C^+^ monocyte recruitment to the steady state gut are, however, still unknown. While they are likely related to the microbiota exposure of the tissue, analysis of germ-free animals has yielded conflicting results (29, 34, 44, 45). The latter could be due to intestinal embryo-derived macrophages that might persist in the absence of arising competition by an adult monocyte influx.

## Macrophage Heterogeneity

Interestingly, emerging evidence suggests that intestinal macrophages are more heterogeneous than previously thought. Monocyte-derived CD11b^+^ CX_3_CR1^+^ cells in the gut comprise both CD11c^+^ and CD11c^−^ cells. While differential functions of these cells remain to be established, studies into this matter might profit from the recent finding that generation of CD11c^+^, but not CD11c^−^ CX_3_CR1^+^ intestinal macrophages requires Notch signaling (21). A subpopulation of CD169-expressing CX_3_CR1^+^ macrophages has been reported to be associated with the intestinal crypts (22), although these cells will require further functional characterization. Bogunovic and colleagues recently reported an intriguing CX3CR1^+^ macrophage subpopulation that resides in the muscularis layer and communicates with enteric neurons to regulate gastrointestinal motility (23). Importantly, we and others have recently shown that macrophages isolated from distinct tissues, such as the liver, lung, brain, and peritoneum, differ considerably with respect to their gene expression profile (46, 47). As expected, this diversity is also prominently reflected in the differential enhancer usage of these cells, as inferred from highly divergent histone modifications (47). Moreover, given that the number of regulatory elements by far exceeds the number of genes (48, 49), this heterogeneity is even more pronounced, including both active and poised enhancer states (47). This applies, albeit to a lesser extent, also to macrophages located in proximal and distal segments of the gut (47). Epigenetic heterogeneity of intestinal macrophages likely reflects monocyte exposure to distinct environmental cues in ileum and colon during their local differentiation (32, 47). In-depth understanding of how these macrophage identities are established, including the hierarchy of induced transcription factors, could yield valuable insights into monocyte differentiation that might be applicable to other tissues and inflammatory settings. PU.1 is a pioneering factor, which induces c-fms transcription and is hence required for macrophage differentiation (50). Intestinal macrophages are furthermore characterized by prominent expression of the Runt-related transcription factor 3 (Runx-3) (47). Interestingly, mice that harbor Runx3 deficiency develop spontaneous colitis (51). Other candidates that might be involved in the establishment of the intestinal macrophage signature are the interferon regulatory factors 4 and 5 (Irf-4, Irf-5), shown to be associated with classical and alternative macrophage activation, respectively (52–54).

## Macrophage Interactions with Their Environment

### Macrophage communication with the epithelial cell layer

Pioneering studies by Rescignio and colleagues revealed that certain intestinal mononuclear phagocytes can penetrate the intestinal epithelium by virtue of expression of tight junction proteins and formations of dendritic projections (55). These structures, later termed trans-epithelial dendrites (TEDs) (56), were subsequently ascribed to macrophages expressing CX_3_CR1 (18) and allegedly allow these non-migratory cells to sense, and potentially sample, the luminal content (18, 56). TED formation by macrophages in the terminal region of the ileum was found to be dependent on expression of both CX_3_CR1 macrophages and its membrane-tethered ligand CX_3_CL1/Fractalkine by selected epithelial cells (57). CX_3_CR1-deficient and CX_3_CL1-deficient mice were reported to be relatively protected from acute, DSS-induced colitis (58) – a phenotype that might be related to TED formation (57). Likewise, CX_3_CR1-deficient mice were shown to display impaired oral tolerance, which was related to impaired IL-10 production by intestinal macrophages, though not their TED formation (59). Finally, there is evidence for a potential role of CX_3_CR1^+^ macrophages in the capture of luminal bacteria (60) and even the transport of the latter to lymph nodes, at least under conditions of dysbiosis (61). However, the exact definition of macrophage contributions in their native tissue context remains challenging, because it requires their accurate discrimination from closely related and phenotypically similar monocyte-derived DC.

Apart from their role in maintaining intestinal immune homeostasis, gut macrophages also contribute critically to epithelial wound healing. Macrophages associated with the crypts of Lieberkuehn in the colon were reported to assist, following tissue damage, the proliferation and survival of epithelial progenitor cells in a Myd88-dependent manner (62–64). Moreover, in a murine model of acute epithelial regeneration in the colon, activated macrophages supported tissue repair by up-regulating expression of IL-3 and IL-4, while inhibiting secretion of TNF and interferon-γ (IFN-γ) in the lamina propria (3, 65). Macrophages also appear to be able to influence the permeability of the epithelium barrier via the secretion of IL-6 and NO, thereby potentially increasing the invasion of pathogens (66).

## Communication with Immune Cells

Macrophages are inferior to DC in their ability to prime naïve T cells (67). This might be due to their rapid degradation of ingested proteins, which impairs their ability to retain antigens for presentation (68). Moreover, at least in steady state, intestinal CX_3_CR1^+^ macrophages lack expression of CCR7, i.e., the chemokine receptor required for migration to the MsnLNs (25, 69). Rather, the cells that reside in the lamina propria have been proposed to maintain the functionality of FoxP-3^+^ T regulatory cells that migrated back from the MsnLNs into the tissue (59). Thus, while Treg cell generation of CX_3_CR1-deficient mice is unimpaired, these animals harbor reduced Treg cell numbers in the lamina propria, a phenotype that is associated with impaired oral tolerance (59). In light of other data (70), the authors of this study linked the reduced FoxP-3^+^ Treg cell numbers to impaired production of IL-10 by CX_3_CR1^+^ macrophages (59). However, the latter might have to be revised, since CX_3_CR1^Cre^:IL10^fl/fl^ mice were shown to harbor unimpaired FoxP-3^+^ Treg cell numbers (35). Also, interactions between CX_3_CR1^+^ macrophages and Th17 cells, which are rarely found in intestinal lymphoid tissues and, though primed in the MsnLN, might terminally differentiate in the lamina propria, remain incompletely defined. On one hand, it was shown that intestinal CD70^hi^ CX_3_CR1^+^ macrophages are activated by commensal-derived ATP and drive the *in vitro* differentiation of Th17 cells (71, 72). On the other hand, intestinal macrophages were reported to counteract Th17 generation that is promoted by CD103^+^CD11b^+^ DC (73, 74). Of note, CD103^+^CD11b^+^ DCs and Th17 cells co-localize in the intestinal tract, as the number of both cells drop from the duodenum to the ileum, and they are scarce in the colon. By contrast, CX_3_CR1^+^ macrophages and FoxP3^+^ Treg cells are most abundant in the colon (74).

Recent findings revealed an intriguing cross-talk between intestinal macrophages and innate lymphoid cells (ILC). Thus, in response to luminal stimuli and using a signaling pathway involving the TLR adaptor Myd88, macrophages were shown to secrete IL-1β and in turn induce production of csf-2 by RORγt^+^ type 3 ILC (20). Mice lacking Csf-2 display reduced numbers of colonic macrophages and DC, associated with a hampered Treg cell compartment (20). Moreover, in a *Citrobacter* infection model CX_3_CR1^+^ macrophages were shown to promote ILC production of IL-22 via secretion of IL-23 (75), in line with another report (76). Interestingly, CX_3_CR1^+^ macrophage-derived IL-23 not only induces IL-22 but also seems to concomitantly suppress IL-12 production by CD103^+^ CD11b^−^ DC and thereby prevents otherwise detrimental immunopathology (77). Notably, the latter finding provides first evidence for the existence of a direct cross-talk among intestinal mononuclear phagocytes in tissue context, a topic that clearly deserves further study.

## Intestinal Dendritic Cells

Dendritic cells are specialized in communicating with T cells, curbing autoreactivity and activating T cell immunity in response to threats. Specifically, DC provide T cells with antigenic peptides that are presented in MHC context, co-stimulation and instructing cytokines that govern T cell polarization into effector cells (67). In order to maintain homeostasis and avoid inflammatory responses toward innocuous antigens, gut DC employ tolerogenic mechanisms that allow them to dampen adaptive immunity. MsnLN- and lamina propria-resident CD103^+^ DC secrete, for example, retinoic acid (RA) and transforming growth factor-β (TGF-β), which promote the generation of Foxp3^+^ Treg cells and contribute to the differentiation of plasma cells, which secrete IgA (78, 79).

## Classification and Ontogeny

Intestinal DC in mice are characterized by the surface expression of the integrins CD11c (α_X_) and CD103 (α_E_β7) (11, 19, 69). More recently, CD24 and Sirpα have been introduced for the better discrimination of DC from macrophages (8, 10). CD103^+^ DC in the gut arise from dedicated DC precursors, or preDC, and accordingly, mice deficient for fms-related tyrosine kinase-3 receptor (Flt-3) or its ligand Flt-3L have significantly decreased levels of intestinal DC (7, 19). Other, currently though less well-characterized DC progenitors are α4β7^+^ so-called “pre-μDC,” which are generated in the bone marrow and were shown to give rise to classical CD103^+^ DC and CCR9^+^ plasmacytoid DC (80).

Classical CD103^+^ DC are divided into two major subpopulations according to their expression of CD11b (α_M_) (81). CD103^+^ CD11b^+^ DC and CD103^+^ CD11b^−^ DC display distinct abundance in small and large intestine, present different additional surface markers, and require different growth factors for their development (82, 83).

CD103^+^ CD11b^+^ DC are developmentally related to CD11b^+^ CD8α^−^ splenic DCs (15) and found in the lamina propria of the small and large intestine. They can migrate in CCR7-dependent manner (84) to the MsnLNs, where they present luminal antigens to T cells. CD103^+^ CD11b^+^ DCs likely represent a heterogeneous population, as a fraction of them is Csf-2-dependent (3). Development of CD103^+^ CD11b^+^ DC, but not of CD103^+^ CD11b^−^ DC, is hampered in Csf-2R-deficient mice (85) and when expression of Notch-2 (12, 76) or IRF-4 (14) is impaired. Moreover, CD103^+^ CD11b^+^ DC numbers are also reduced in absence of RA and under conditions of vitamin A deprivation (15).

CD103^+^ CD11b^−^ DC are more prevalent in lymphoid organs – the Peyer’s Patches, MsnLNs, and ILFs (7, 69). However, they can be found also in animals lacking these structures, and are hence not limited to lymphoid tissues (3). Similar to classical CD8α^+^ DC in the spleen, CD103^+^ CD11b^−^ DC depend on the expression of the transcription factors BatF-3 and Irf-8 (6, 15). Like the former, they also express the chemokine receptor XCR1 that has emerged as a universal marker for this DC subset in mouse and human (8, 9). The connection between CD103^+^ CD11b^−^ DC and CD8α^+^ DC is also supported by the fact that the number of CD103^+^ CD11b^−^ DC was shown to increase, alongside with splenic CD8α^+^ DC, in mice that display constitutive β-catenin activation (86). Moreover, like splenic CD8α^+^ DC (87), also CD103^+^ CD11b^−^ DC are specialized in cross-presentation (88).

The exact definition of intestinal DC is complicated, since monocyte-derived cells can acquire phenotypic and functional DC hallmarks. Studies have described a population of CD103^−^CX3CR1^+^CD11b^+^ DC, which resides in the lamina propria (11, 16). These cells are CSFR-1 dependent and appear to be derived from Ly6C^high^ monocytes (11). Recent studies also reported that under inflammatory conditions, these CD103^−^CX3CR1^+^CD11b^+^ DC expressed CCR7 and migrated in the intestinal lymph, similar to classical intestinal DC, and induced the differentiation of IL-17 and IFN-γ producing T cells (16, 17).

## Antigen Sensing and Uptake

CD103^+^ DC, present in the lamina propria and associated with the intestinal epithelium lining the villi, provide surveillance of the luminal environment (30). They detect foreign and inflammatory signals, acquire and present antigens and interact with T cells by migrating to secondary lymphoid organs (3). Located deep in the core of the villous lamina propria, CD103^+^CD11b^+^ DC would seemingly have limited access to luminal signals, unless antigens or bacteria cross the epithelium or are imported into the lamina propria by other cells, e.g., macrophages, epithelial M cells, or small intestine goblet cells (36, 89, 90). However, lamina propria-resident CD103^+^ DC were shown to migrate into the epithelial cell layer and capture bacterial antigens (90).

## DC Migration

Mucosal T cell priming, arguably one of the primary roles of gut DC, is believed to be restricted to lymphoid tissues (3). Intestinal DC are hence bound to migrate from the lamina propria to the MsnLNs, or within Peyer’s Patches into T cell zones. Indeed, CD103^+^ DC were detected in the intestinal lymph under homeostatic conditions (69, 84). In addition, after systemic BrdU administration, labeled CD103^+^ DC were found in the lamina propria before they could be discerned in the MsnLNs (30). LN-resident CD103^+^ DC are thus derived from the tissue and constantly immigrate (30, 91). Interestingly, steady state migration of intestinal CD103^+^ DC does not appear to be induced by the microbiota or by TLR signaling (92), but may rather depend on a low, tonic release of inflammatory cytokines, or result from spontaneous DC maturation. Nevertheless, entry of CD103^+^ DC into the MsnLNs is of course considerably enhanced by pro-inflammatory cytokines or TLR ligands (93, 94). Migration of intestinal DC depends on CCR7, both in steady state and under inflammatory conditions. Accordingly, CCR7 expression is up-regulated in DC before their migration from the tissue into the MsnLN (84) and CCR7 deficient DC fail to migrate (69, 84, 95). Moreover, it was recently shown that DC can also migrate from the lamina propria into the epithelial layer (90) and can thus gain direct access to antigen and luminal bacteria. Hence, following challenge with *Salmonella*, accumulation of the bacteria was first observed in DC of the epithelial fraction and only subsequently in DC in the lamina propria (90).

## DC and the Epithelium

DC intimately interact with the epithelial layer of the intestine by a variety of mechanisms. Small intestinal goblet cells were shown to transfer small soluble antigens from the intestinal lumen to CD103^+^ DC (89). Chemokines secreted by enterocytes in response to TLR ligand exposure can induce the above-mentioned relocation of lamina propria DC to the epithelium (90). In addition, it is becoming more and more evident that epithelial cells play a critical role in maintaining DC in a tolerogenic state, compatible with gut homeostasis. Epithelial and stromal cells secrete factors, which are thought to induce DC tolerance, such as RA, TGF-β, PGE-2, and TSLP (3, 82, 96–99). In parallel to ILC (20), intestinal epithelial cells regulate retinal dehydrogenase (RALDH) expression by CD103^+^ DC that the cells need to metabolize retinoids. Specifically, epithelial cells express a critical cytosolic retinoid chaperone, the cellular retinol binding protein II, which is required for *in vivo* imprinting of gut DC by lumenal retinoids (99, 100). Supporting this notion, the *in vitro* co-culture of bone marrow- or spleen-derived DC with epithelial cells results in the up-regulation of CD103 and RALDH, together with TGF-β imprinted homing potential on T cells (101–103). These data establish the potential of intestinal epithelial cells to educate intestinal DC, although further *in vivo* studies and higher resolution, with respect to cell subsets, are required to better elucidate the underlying mechanisms.

## DC Communication with Intestinal T Cells

Intestinal CD103^+^ DC, found in lamina propria, Peyer’s Patches, and the MsnLNs program T cells to express the gut-homing factors CCR9 and α4β7 integrin (101, 104, 105). Concomitantly, DC can also induce the development of FoxP-3^+^ and IL-10 producing Treg cells (106) and prime Th17 cells (17, 107, 108). The majority of these DC-governed priming events require TGF-β signaling and RA, which are generated in the DC by enzymatic conversion of all-trans-retinal, a derivative of vitamin A, using RALDH2 (101, 109, 110). Indeed, RA has emerged as the critical conditioning factor for intestinal DC, as vitamin A is crucial for the activity of the enzyme RALDH in DC. Without RALDH, the ability of DC to imprint T cells is hampered, and restored only after vitamin A administration (111). The balance between RA and TGF-β levels seems to determine the fate of Treg cells primed by DC, as presence of both RA and TGF-β favor the development of FoxP-3^+^ cells, while RA induces the generation of IL-10 producing T cells (106).

Other enzymes that influence the outcome of T cell priming are indoleamine 2,3 dioxygenase (IDO) and TSLP. IDO is expressed also by DC in other tissues and was shown to inhibit the development of effector T cells and promote Treg cell generation (112, 113). TSLP is, as mentioned above, secreted by epithelial cells, but also by the intestinal DC, themselves. In the presence of TSLP, Th17 responses are restricted due to a reduced ability to produce IL-17, and Treg cell differentiation is up-regulated (107). The ability of the intestinal DC compartment to generate Th17 cells seems to be associated with CD103^+^ CD11b^+^ DC, as the frequency of Th17 cells is reduced in mice lacking these DC due to either IRF-4 or Notch-deficiency (10, 12, 14), or as a result of conditional ablation of this DC subset (13). Interestingly though, a recent study showed that also another subpopulation of DC, i.e. CCR2^+^ CD103^−^ CD11b^+^ DC can induce IL-17a production in CD4^+^ T cells and effectively prime Th17 cells, probably via IL-12/IL-23p40 secretion (17).

## Intestinal DC, Inflammation, and Immune Response

In steady state, intestinal DC are probably mainly tolerogenic. Under inflammatory conditions, however, they can become highly effective T cell activators (114). Induction of experimental colitis results in the accumulation of CD103^+^ DC with an inflammatory profile in the MsnLNs (114). These DC express less RALDH and TGF-β and instead of promoting Treg cell formation, now induce Th1 inflammatory responses (114). While Th17 polarization might be carried out by CD103^+^ CD11b^+^ DC (12), differentiation of CD8^+^ effector T cells under inflammatory conditions seems to be dependent on CD103^+^ CD11b^−^ CD8α^+^ DC that migrated into the lymph (88).

Flagellin stimulation causes TLR-5^+^ CD103^+^ DC in the small intestine to promote differentiation of Th17 cells and secrete IL-23, which in turn induces IL-22 production by ILC3 and subsequent epithelial up-regulation of antibacterial peptides (115).

In summary, DC are major players in maintaining homeostasis in the intestine. While tolerogenic at steady state, under inflammatory conditions they tip the scales and activate the immune system. They can migrate between different compartments of the intestine – from the lamina propria to the epithelium and into the MsnLNs – and execute different immune responses in each tissue. Further research regarding the location of DC, their functions and characteristics should shed new light on the role of these cells in the intestine.

## Concluding Remarks and a Glimpse to the Future

**Figure 1:**
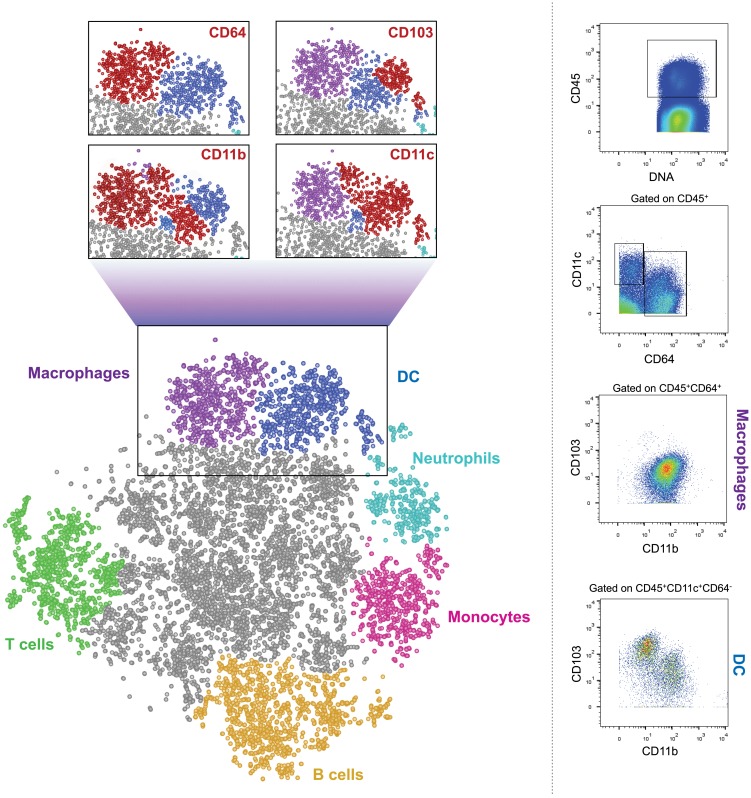
**CyTOF analysis of CD45^+^ cells from murine colon**. Cells were isolated from colon of 6–9 weeks old WT female C57Bl/6 mice and stained with a panel of 26 cell surface markers. The results were gated for live, single, CD45^+^ cells. Bh-SNE analysis and clustering were performed by Accense (http://www.cellaccense.com/) and the results were processed by GIMP. Colors indicate high levels of the following markers: green – TCRβ, CD3e (T cells), Orange – B220 (B cells), light blue – Ly6G (granulocytes), pink –Ly6C (monocytes), purple – CD64, F4/80 (macrophages), blue – clustered by Accence, different DC populations, gray – non-identified or non-specific cells. Red populations in zoom-in black squares indicate high levels of the marker written. Representative of at least four separate, independent experiments.

In summary, macrophages and DC critically contribute to intestinal homeostasis and immune defense. Both cellular compartments have been subdivided into discrete subpopulations, which though currently mainly phenotypically defined, in some cases have been assigned distinct activities. The challenge ahead is to better define precise roles of these subsets both in health and under inflammatory conditions, first in the mouse but then also in the human. This task is complicated by the fact that many of the used markers used to distinguish between subpopulations of DC and macrophages are shared by the two types of mononuclear phagocytes. Moreover, under inflammatory conditions monocyte-derived cells further blur the picture. Collectively, this highlights the need to define cells by multiple parameters, including both surface and intracellular markers. Single cell transcriptome analysis is likely to help with this task (116, 117). However, classic flow cytometry analysis using fluorescent dye-coupled antibodies allows only a very limited simultaneous panel of markers due to the few dyes available and the spectral overlap of their emission. This problem might, in the near future, be solved by spectral cytometry systems that use ultrafast optical spectroscopy combined with flow cytometry to differentiate between the emission curves of different fluorophores, thus enabling the use of dozens of antibodies in one sample (118). Moreover, a new cell analyzer has been introduced, which uses mass cytometry instead of flow cytometry and is termed cytometry by Time-Of-Flight, or CyTOF (119). Instead of conjugations to fluorophores, this machine uses conjugations to heavy metal isotopes. Such metals do not exist naturally in the cells, so background is insignificant. The stained cells are injected into the CyTOF and are evaporated in a plasma chamber. The metals are ionized, hit the TOF detector, and their mass is measured, allowing the machine to determine the expression levels of the markers on each cell. This multiple-parameter approach enables to explore entire immune cell populations and subpopulations from the same tissue. As exemplified in Figure 1, such global analysis methods might well hold the key for the better definition and understanding of the cellular make-up of the intestine. No doubt, that with the recent development in the fields of cell cytometry and RNA sequencing, more pieces of this complex puzzle of the characteristics and roles of mononuclear phagocytes in the gut will be detected and put in place.

## Conflict of Interest Statement

The Guest Associate Editor Martin Guilliams declares that, despite having collaborated on a paper with Steffen Jung in May 2013, the review process was handled objectively. The authors declare that the research was conducted in the absence of any commercial or financial relationships that could be construed as a potential conflict of interest.
